# Mendelian randomization and single-cell expression analyses identify the causal relationship between depression and chronic rhinosinusitis

**DOI:** 10.3389/fpsyt.2024.1342376

**Published:** 2024-05-16

**Authors:** Fangwei Zhou, Yan Yang, Jianyao Li, Ying Jin, Tian Zhang, Guodong Yu

**Affiliations:** Department of Otorhinolaryngology Head and Neck Surgery, Affiliated Hospital of Guizhou Medical University, Guiyang, China

**Keywords:** chronic rhinosinusitis, depression, causality, Mendelian randomization, single-cell

## Abstract

**Background:**

The causative relationship between chronic rhinosinusitis (CRS) and depression remains unclear. Herein we employed Mendelian randomization (MR) coupled with single-cell analysis to investigate the causality between CRS and depression.

**Methods:**

Data pertaining to CRS and depression were mined from the genome-wide association study database, and a single-cell dataset was sourced from the literature. To explore causality, we conducted bidirectional MR analysis using MR-Egger, weighted median, inverse variance weighted (IVW), simple mode, and weighted mode, with IVW representing the most important method. Further, sensitivity analysis was performed to evaluate the robustness of MR analysis results. Candidate genes were analyzed via single-cell combined MR analysis.

**Results:**

Forward MR analysis indicated depression as a risk factor for CRS when depression was the exposure factor and CRS was the outcome (OR = 1.425, *P* < 0.001). Reverse MR analysis revealed the same positive relationship between CRS and depression when CRS was the exposure factor and depression was the outcome (OR = 1.012, *P* = 0.038). Sensitivity analysis validated the robustness of bidirectional MR analysis results. Ten cell types (endothelial, ciliated, basal, myeloid, mast, apical, plasma, glandular, fibroblast, and T cells) were identified in the single-cell dataset. The network of receptor–ligand pairs showed that in normal samples, cell–cell interactions were present among various cell types, such as epithelial, mast, myeloid, and endothelial cells. In contrast, CRS samples featured only one specific receptor–ligand pair, confined to myeloid cells. *TCF4* and *MEF2C* emerged as potentially crucial for CRS-associated depression development.

**Conclusions:**

Our findings suggest a bidirectional causal relationship between CRS and depression, offering a new perspective on the association between CRS and depression.

## 1 Background

Chronic rhinosinusitis (CRS) is one of the most prevalent chronic nasal diseases characterized by prolonged inflammation of the nose and paranasal sinus, with symptoms persisting for more than 12 weeks (1). The main symptoms of CRS include nasal obstruction, nasal discharge, olfactory dysfunction, and facial pain/pressure (2). Furthermore, CRS is evidently associated with several non-sinonasal symptoms, including cognitive dysfunction, sleep dysfunction, and significantly low productivity levels (3). Its global prevalence is estimated to be >10%, with varying rates in different regions, such as approximately 5% in Canada, 7% in South Korea, 11% in Europe, 12% in the United States, and 13% in China (4, 5). The diagnosis of CRS is a comprehensive process, typically relying on clinical symptoms, imaging results, and nasal endoscopy, all evaluated using globally recognized criteria. Variations or broader diagnostic standards may result in disparities in the reported prevalence of CRS among various studies. The economic burden of CRS remains substantial, with direct costs ranging from 10 to 13 billion dollars annually and indirect costs exceeding 20 billion dollars (6). Some patients continue to experience symptoms despite optimal medical treatments and appropriate surgical interventions, which can be challenging. In such cases, subjective symptoms of sinusitis persist even when radiologic or endoscopic evidence suggests improvement, and these symptoms may be influenced by various factors, such as mental and physical capabilities (7).

A recent cohort study revealed a significant association between CRS and an increased risk of depression, indicating that patients with CRS are 1.51 times more likely to develop depression compared to the general population (8). Depression is a prevalent disorder that not only significantly affects patient quality of life and productivity but also amplifies the perceived burden of chronic illnesses. Moreover, depression can influence the degree of improvement achieved following surgical interventions (9). Notably, the coexistence of depression with other health conditions often heightens the risk of adverse health outcomes (10). Comorbid depression, unfortunately, frequently goes undiagnosed in patients with chronic disorders, including CRS. This could be attributed to the fact that healthcare providers primarily focused on treating the primary chronic condition might not give adequate attention to or recognize the significance of underlying depression. Accurate diagnosis of comorbid depression may also serve as an important prognostic indicator for CRS treatment outcomes. However, the precise relationship between depression and CRS is still largely unknown.

Mendelian randomization (MR) is a powerful tool to investigate causal associations by employing common genetic variants as unconfounded and unbiased proxies (11). Unlike observational studies, MR is a statistical approach that utilizes single nucleotide polymorphisms (SNPs), which remain independent of confounding factors due to the random allocation of alleles from parental to filial generations and the unidirectional flow from genotype to phenotype. These SNPs function as instrumental variables (IVs) to evaluate the causal relationship between an exposure and an outcome (12). Genome-wide association studies (GWAS) focused on diverse complex traits have emphasized that genetic variants are notably enriched in non-coding regions with cis-regulatory activities, and these regions are further enriched in expression quantitative trait loci (eQTL) (13). By combining genetic data with bulk RNA sequencing, downstream effects of genetic risk factors associated with diseases have been linked to eQTL (14). This novel analytical framework, which integrates eQTL and GWAS data, has been successful in determining gene expressions that are pleiotropically or potentially causally linked to different phenotypes. This approach holds promise as a prospective tool to explore genes with pleiotropic associations with complex traits (15).

Despite its relevance to various chronic disease processes, depression has not been thoroughly investigated as a risk factor for CRS. Considering the existing uncertainty regarding the causal relationship between depression and CRS, herein we first performed a bidirectional MR analysis that integrated GWAS data on depression and CRS to evaluate the causal relationship between these conditions. Besides, we performed single-cell analyses of CRS in conjunction with MR to investigate the role of SNPs in gene expression in a single-cell dataset. Our findings hold significant implications for enhancing our understanding of the causal relationship between depression and CRS. This knowledge can aid healthcare providers and policymakers in designing more effective treatment and management strategies for CRS.

## 2 Methods

### 2.1 Data source

We downloaded the finn-b-J10_CHRONSINUSITIS dataset for CRS, containing 16,380,288 SNPs from 176,373 samples, and the ieu-b-102 dataset for depression, containing 500,199 samples, from the IEU Open GWAS database (https://gwas.mrcieu.ac.uk/). A single-cell dataset for CRS was sourced from the literature (16).

### 2.2 Data pre-processing

Exposure factors were extracted and filtered using the “extract instruments” function of the TwoSampleMR package (17), with P < 5×10^−8^ for forward and *P* < 5×10^−5^ for reverse MR analyses. The initial analysis indicated that, using a strict threshold (*P* < 5×10^–8^), the number of IVs was exceedingly limited. Thus, we adopted a less restrictive threshold (*P* < 5×10^–5^) to acquire a broader range of IVs, striving for comprehensive outcomes. IVs in linkage disequilibrium were removed to ensure independence, with the criteria set at r^2^ = 0.001 and kb = 10000 for both forward and reverse MR analyses. We retained IVs with strong correlations with exposure factors. Three fundamental assumptions underlie MR studies (18): (1) a robust and noteworthy relationship between IVs and exposure; (2) IVs are unrelated to confounding factors; and (3) IVs exclusively influence outcomes through exposure, not through other channels.

### 2.3 Bidirectional MR analysis

Following IV filtering, we obtained input data for both forward and reverse MR analyses. We conducted bidirectional MR analysis using MR-Egger, weighted median, inverse variance weighted (IVW), simple mode, and weighted mode, with IVW being the most important method (19). For our analysis, odds ratio (OR) > 1 indicated a risk factor and OR < 1 indicated a protective factor. Scatter, forest, and funnel plots were used for result visualization.

### 2.4 Sensitivity assessment of bidirectional MR analysis

To assess the reliability of our forward and reverse MR analysis results, we performed sensitivity analysis. First, we conducted a heterogeneity test, with Q_pval > 0.05 indicating no heterogeneity. Next, we performed a horizontal pleiotropy test using the TwoSampleMR function “mr_pleiotropy_test” in R; *P* > 0.05 indicated no horizontal pleiotropy, implying no confounding factors. Finally, we implemented the leave-one-out (LOO) method by iteratively excluding each SNP. If the effect of the remaining SNPs on the outcome variable did not markedly change, it indicated the reliability of the MR analysis results.

### 2.5 Single-cell data analysis

Single-cell analysis was performed using the Seurat package on a single-cell dataset for CRS (with nasal polyps) (20). Before analyzing single-cell gene expression data, we first need to filter out low-quality cells. The purpose of filtering out low-quality cells is to ensure that counting errors do not affect downstream analysis. Cells with too small or too large libraries, or with excessively low or high feature expression levels, are inferred as low-quality cells. Such cells may correspond to dead cells, membrane-damaged cells, or doublets, which can affect downstream analysis. For example, this group of low-quality cells may cluster together and then influence our judgment of cell subpopulations. Low-quality cells typically have smaller libraries, and a normalization of the library size is usually performed before differential analysis. We initiated the analysis by performing quality control on library size and total gene count distribution. Subsequently, highly variable genes in the log-normalized data were retrieved using the “vst” method in the FindVariableFeatures function. Principal components (PCs) were then selected by downscaling analysis using the ScaleData function. Unsupervised cluster analysis of the filtered cells was then performed using the FindNeighbors and FindClusters functions, and the results were visualized using t-SNE and UMAP. We also explored marker cell expression for each cluster in the single-cell dataset and determined the cell type of each cluster. The number of cells and differentially expressed genes (DEGs) in normal and CRS samples were then counted. Cell communication and interactions were analyzed using CellPhoneDB.

### 2.6 Single-cell combined MR analysis

Phenotype-related candidate genes were identified based on single-cell eQTLs and SNPs associated with depression obtained from forward MR analysis. We collected genes with remarkable differences in expression across various cell types, and the expression patterns of these candidate genes were analyzed.

## 3 Results

### 3.1 Depression as a risk factor for CRS

**Figure 1:**
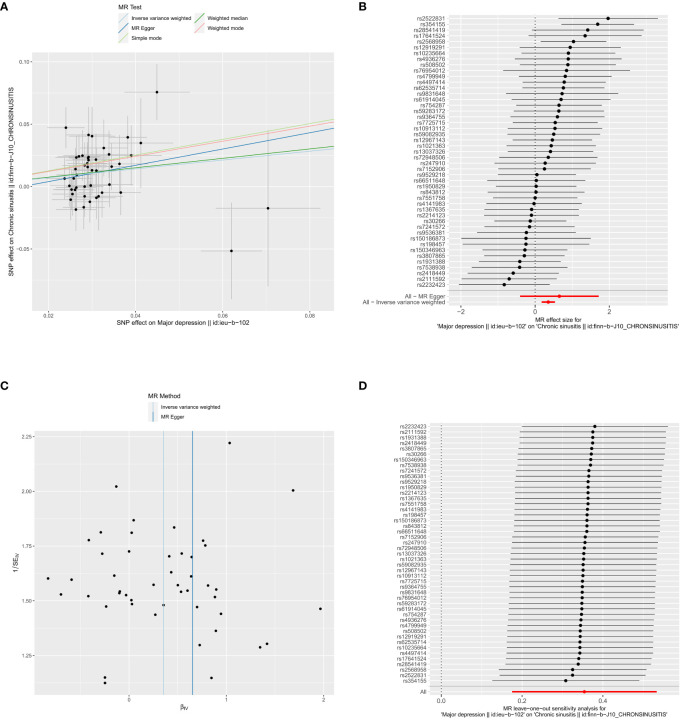
Causal effects of depression on CRS. **(A)** Scatter plot illustrating the association between depression and CRS. **(B)** Forest plot showing the causal effects of depression-associated SNPs on CRS. **(C)** Funnel plot testing the significance of observed associations for heterogeneity. **(D)** Leave-one-out analysis evaluating the effect of each SNP on causality.

We identified 49 SNPs as IVs (**Supplementary Table S1**). Subsequently, we performed forward MR analysis to determine the effect of depression on CRS, with depression being the exposure factor and CRS being the outcome. Our results demonstrated that depression (*P* < 0.001) had a causal association with CRS (**Table 1**) based on IVW, with OR of 1.425, signifying that depression was a risk factor for CRS. The scatter plot showed a positive slope for depression, revealing that depression led to increased risk of CRS (**Figure 1A**). The forest plot showed that all-IVW was on the right, which further supported depression as a risk factor for CRS (**Figure 1B**). Finally, the funnel plot revealed that MR conformed to Mendel’s second law of random grouping (**Figure 1C**).

**Table 1:** Causal effects of depression on CRS.

Exposure	Outcome	Method	P value	OR	OR_lci95	OR_uci95
Depression	CRS	MR Egger	0.233	1.921	0.666364311	5.539574256
		Weighted median	0.003	1.458	1.129336861	1.882524107
		IVW	0.000	1.425	1.191737712	1.704075962
		Simple mode	0.051	1.879	1.013934418	3.48401269
		Weighted mode	0.046	1.832	1.026057373	3.271516154

Sensitivity analysis was performed to determine the reliability of MR analysis results. The heterogeneity test yielded a Q_pval of 0.518, suggesting no heterogeneity among the samples (**Table 2**). Horizontal pleiotropy testing also indicated no horizontal pleiotropic effects (*P* = 0.578) between depression and CRS. Further, LOO analysis results were consistent with IVW analysis results, reinforcing the reliability of our findings (**Figure 1D**). In conclusion, depression was causally related to CRS development, with depression serving as a risk factor.

**Table 2:** Sensitivity analyses of the forward MR study.

Exposure	Outcome	SNP (n)	Heterogeneity tests	Cochran’s Q	Q_ pval	Horizontal Pleiotropy tests	Intercept	P value
Depression	CRS	49	MR Egger	44.602	0.489	MR Egger intercept test	-0.009	0.578
			IVW	44.917	0.518			

### 3.2 CRS as a risk factor for depression

**Figure 2:**
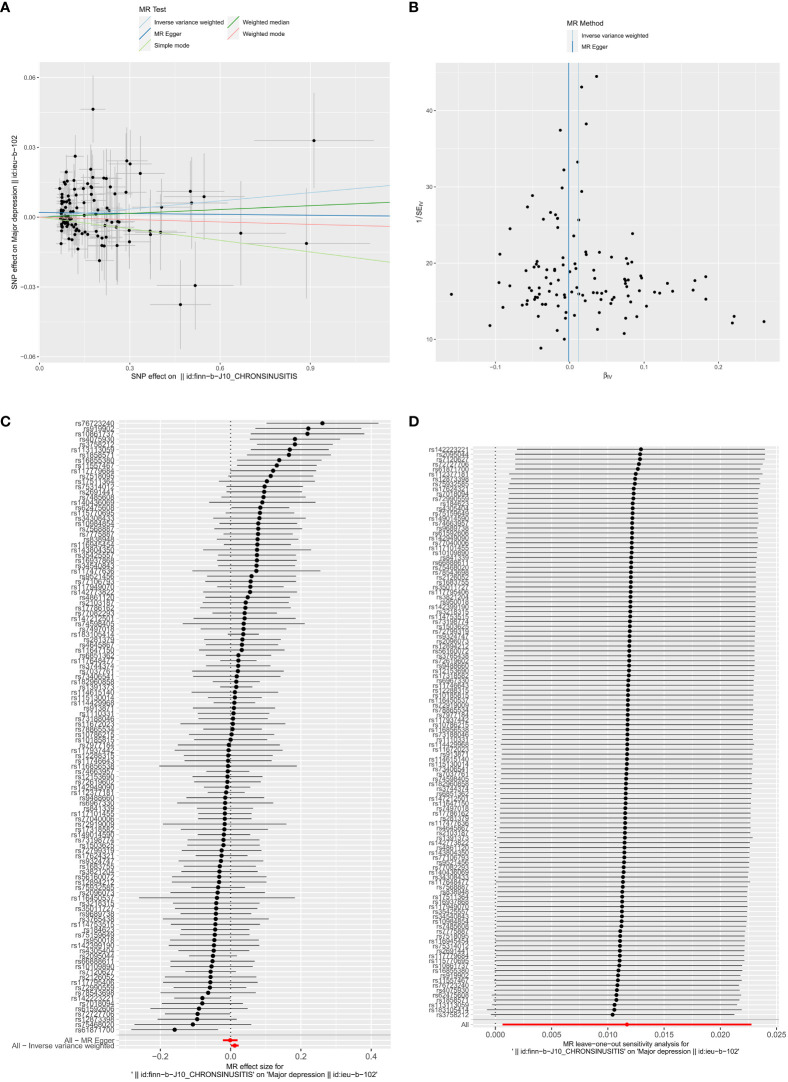
Causal effects of CRS on depression. **(A)** Scatter plot illustrating the association between CRS and depression. **(B)** Funnel plot testing the significance of observed associations for heterogeneity. **(C)** Forest plot showing the causal effects of CRS-associated SNPs on depression. **(D)** Leave-one-out analysis evaluating the effect of each SNP on causality.

Following screening, we identified 117 SNPs as IVs (**Supplementary Table S2**). In reverse MR analysis, with CRS as the exposure factor and depression as the outcome, our results showed that CRS was a risk factor for depression (IVW: *P* = 0.038 and OR = 1.012) (**Table 3**). The scatter plot exhibited a positive IVW slope, indicating an increased risk of depression due to CRS (**Figure 2A**). The funnel plot showed that MR conformed to Mendel’s second law of random grouping (**Figure 2B**). The forest plot indicated that all-IVW was on the right, supporting CRS as risk factor for depression (**Figure 2C**).

**Table 3:** Causal effects of CRS on depression.

Exposure	Outcome	Method	P value	OR	OR_lci95	OR_uci95
CRS	Depression	MR Egger	0.904	0.999	0.978205537	1.019661809
		Weighted median	0.459	1.005	0.991029062	1.020174584
		IVW	0.038	1.012	1.000632919	1.023018792
		Simple mode	0.339	0.984	0.950718662	1.017442879
		Weighted mode	0.774	0.997	0.973659244	1.020045207

Sensitivity analysis for reverse MR also showed promising results. The heterogeneity test yielded a Q_pval of 0.076 and *P* for IVW was >0.05, indicating no heterogeneity among the samples (**Table 4**). Horizontal pleiotropy testing also indicated no horizontal pleiotropic effects between CRS and depression (*P* = 0.151). In addition, LOO analysis results verified that the results were reliable (**Figure 2D**). In summary, CRS was causally related to the occurrence of depression, with CRS leading to an increased risk of depression.

**Table 4:** Sensitivity analyses of the reverse MR study.

Exposure	Outcome	SNP (n)	Heterogeneity tests	Cochran’s Q	Q_pval	Horizontal Pleiotropy tests	Intercept	P value
CRS	Depression	117	MR Egger	161.829	0.076	MR Egger intercept test	0.001	0.151
			IVW	164.797	0.068			

### 3.3 Identification of 10 cell types in normal and CRS samples

**Figure 3:**
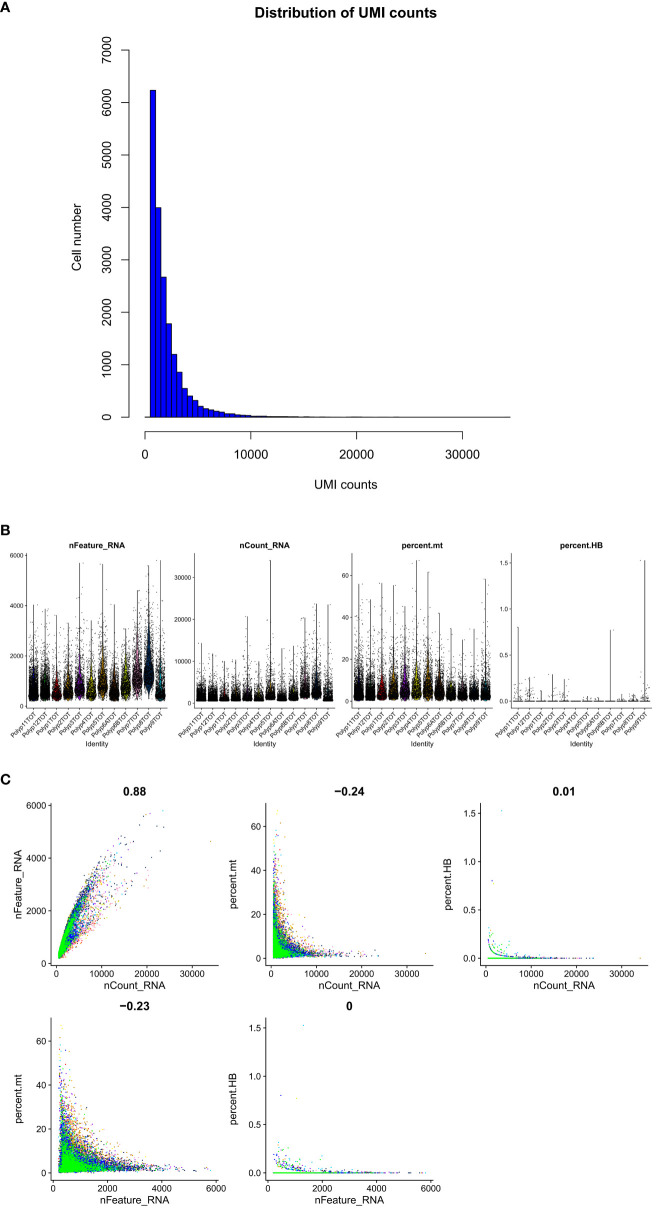
Distribution of library size and total number of genes. **(A)** Distribution of library size. **(B)** Distribution of library size, gene counts, mitochondria percent, and HB percent. **(C)** Distribution of UMI counts, gene counts, mitochondria percent, and HB percent in feature scatter.

**Figure 4:**
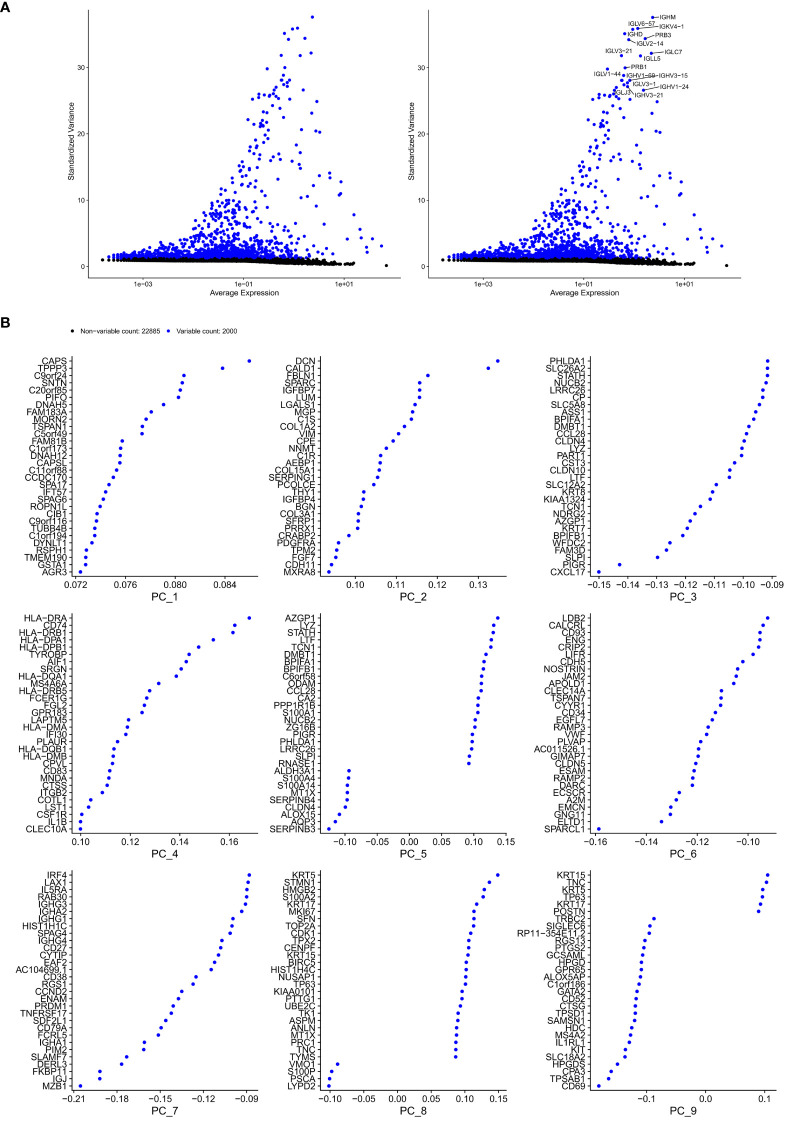
Selection of genes with high variability. **(A)** Highly variable and **(B)** PCA-related genes (top9 dimensions).

**Figure 5:**
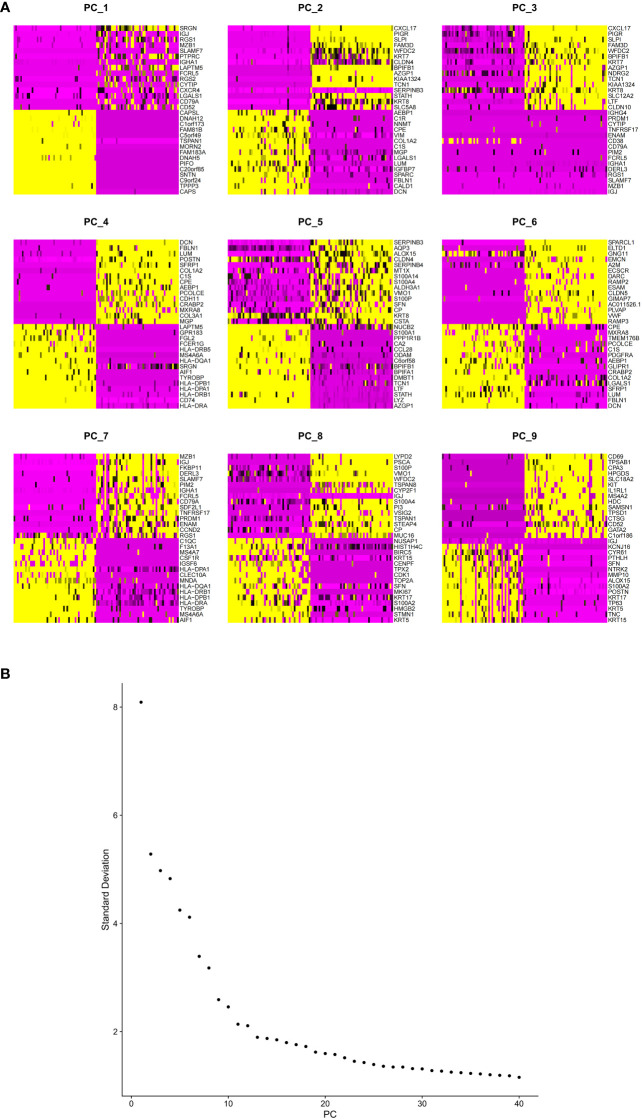
Selection of genes with high variability. **(A)** Heatmap of PCA-related genes (top9 dimensions). **(B)** PCA results with elbow plot.

**Figure 6:**
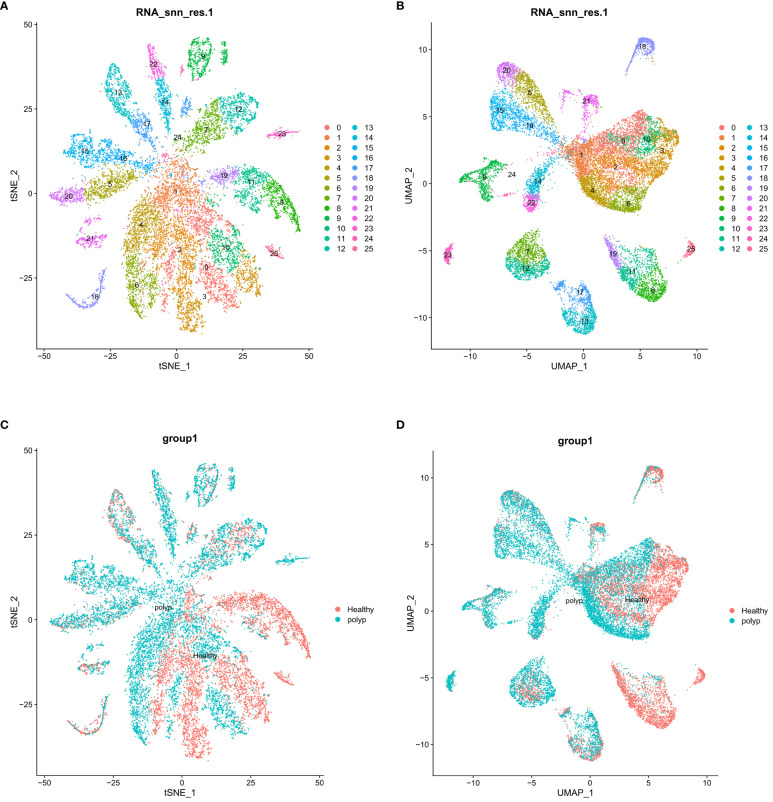
Clustering of cell groups. **(A)** Clustering map of cells (t-SNE). **(B)** Clustering map of cells (UMAP). **(C)** Clustering of cells between groups (t-SNE). **(D)** Clustering of cells between groups (UMAP).

**Figure 7:**
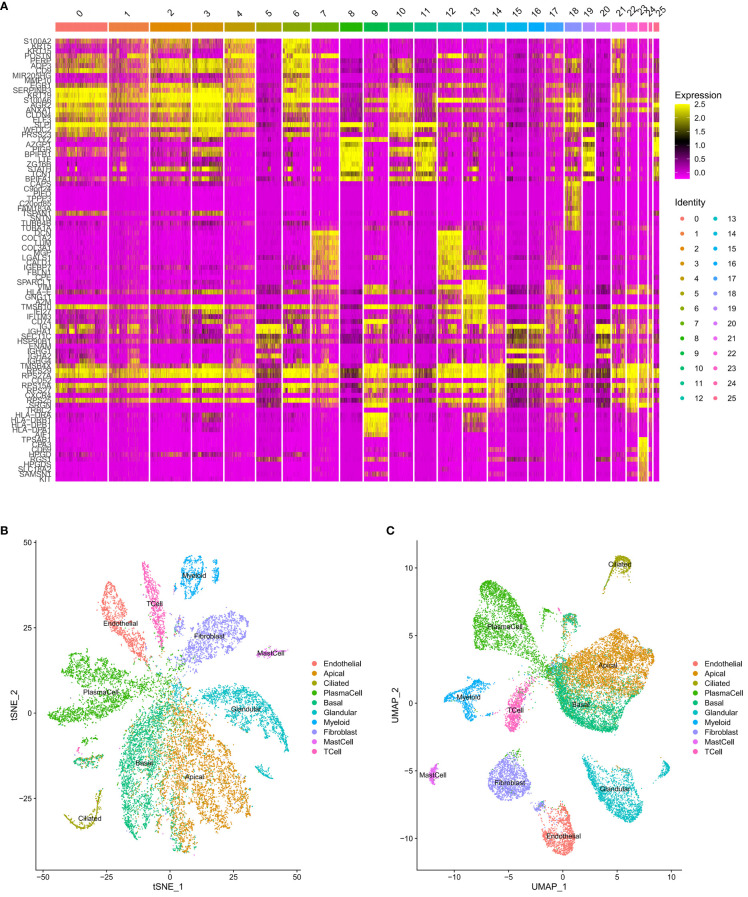
Identification of each cluster cell type. **(A)** Heatmap of genes. **(B)** Clustering map of cells after naming (t-SNE). **(C)** Clustering map of cells after naming (UMAP).

**Figure 8:**
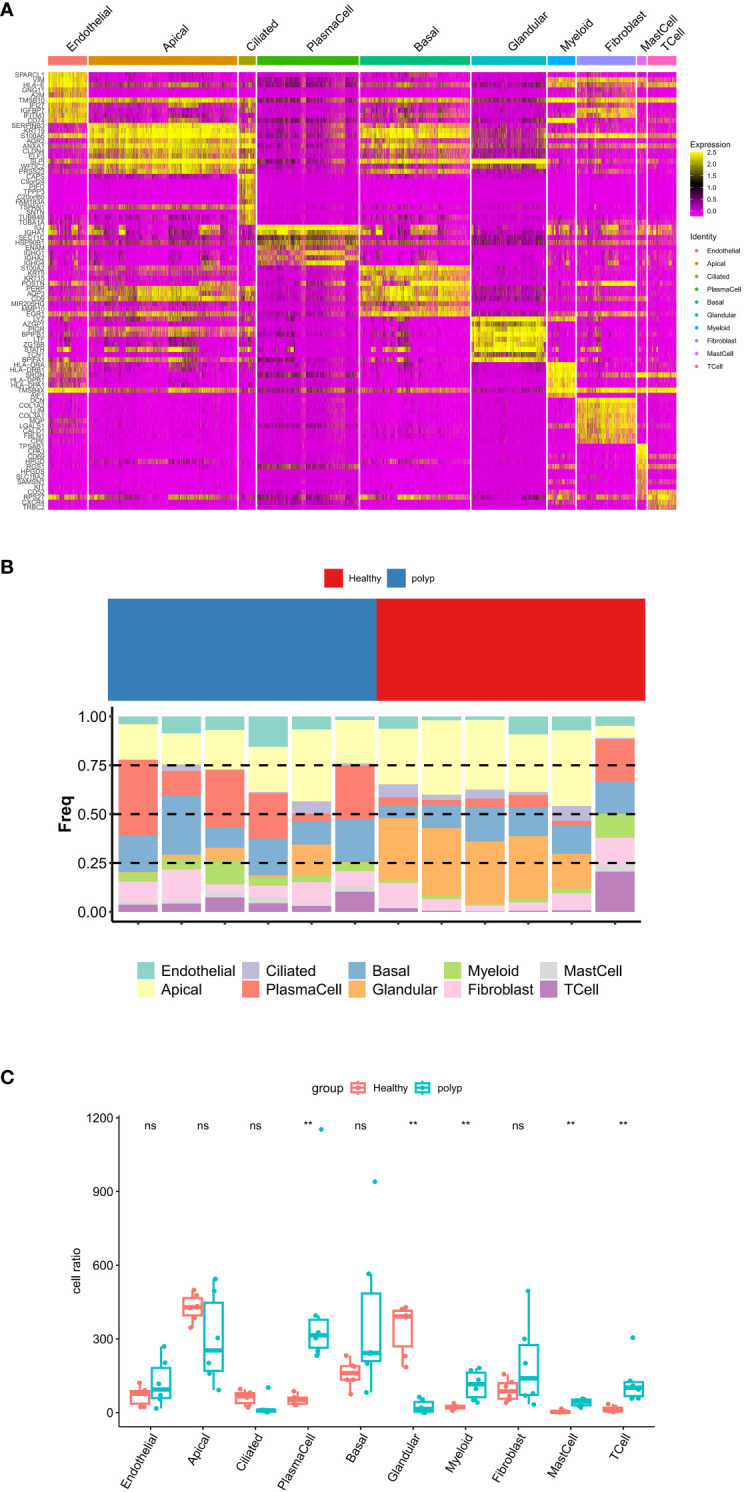
Single-cell analysis of CRS. **(A)** Heatmap of genes. **(B)** Proportional bar chart of cells. **(C)** Proportional boxplot graph of cells. **P < 0.01, ns, no significance.

**Figure 3** shows the characteristics of the single-cell dataset, such as distribution of library size, gene counts, and mitochondria percent. Overall, we identified 2,000 highly variable genes and selected 20 PCs for subsequent analyses (**Figures 4**, **5**). Visualization through t-SNE and UMAP graphs revealed 26 cell clusters in the polyp and healthy samples (**Figure 6**). By evaluating marker gene expression in these clusters (**Figure 7**), we identified 10 cell types: endothelial, ciliated, basal, myeloid, mast, apical, plasma, glandular, fibroblast, and T cells (**Figure 8**). The levels of plasma, glandular, myeloid, mast, and T cells were significantly different between normal and CRS samples (**Figure 8**). We then conducted differential expression analysis to determine the number of significantly DEGs in these 10 cell types, as shown in **Supplementary Table S3**.

The normal samples exhibited a noteworthy presence of 18 specific receptor–ligand pairs, which mediated communication between 45 cell types (**Supplementary Figure S1A**). The network of receptor–ligand pairs between these various cell types in normal samples revealed a rich landscape of cell–cell interactions, involving various cell types, such as epithelial, mast, myeloid, and endothelial cells (**Supplementary Figures S1B, C**). In contrast, CRS samples featured only one specific receptor–ligand pair, confined to myeloid cells (**Supplementary Figures S1D, E**).

### 3.4 Single-cell combined MR analysis revealed 15 candidate genes with remarkable differences in various cell types

**Figure 9:**
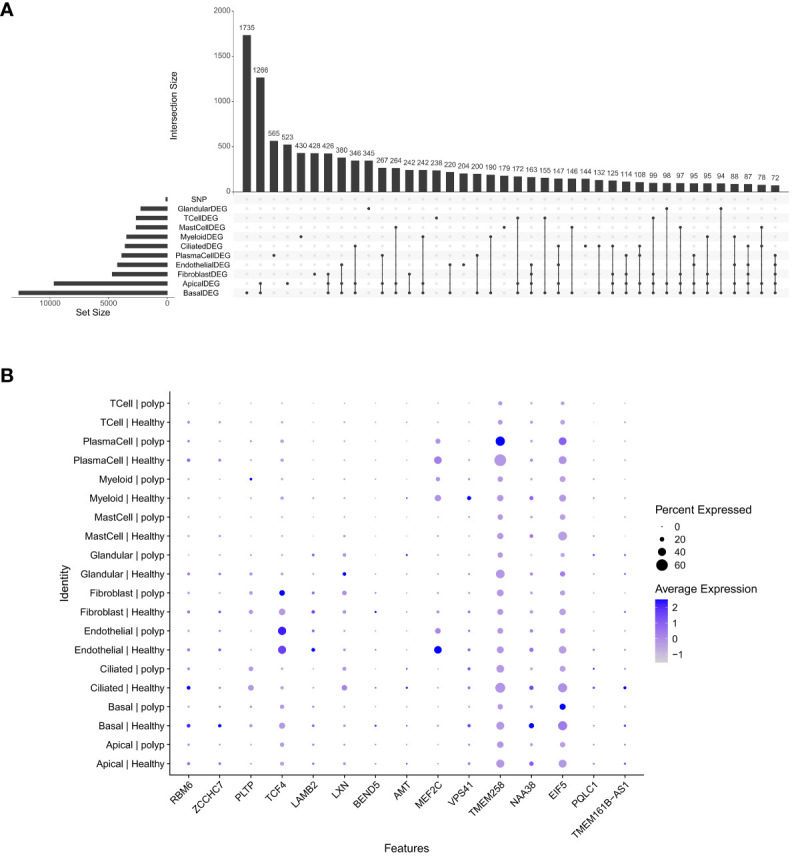
Identification of key genes. **(A)** Upset map of differential genes. **(B)** Bubble map of candidate genes.

Leveraging the 49 SNPs associated with depression from forward MR analysis, we identified 121 phenotype-related candidate genes based on single-cell eQTLs and the GWAS database. These candidate genes were assessed for their expression across 10 cell types (**Figure 9A**). Fifteen of them exhibited significant differences in expression across more than five cell types (**Figure 9B**). *TMEM258*, *NAA38*, and *EIF5* were expressed across almost all cell types, indicating limited cell specificity for these three genes. *TCF4* was more highly expressed in endothelial cells in healthy samples and in fibroblasts in patient samples. *MEF2C* was more highly expressed in endothelial cells in healthy samples. These findings suggested that *TCF4* and *MEF2C* might play a vital in development of CRS with depression.

## 4 Discussion

The prevalence of depression among patients with CRS is high and considerably exceeds the level observed in the general population (21). The exact underlying mechanisms remain unknown. A myriad of non-sinonasal symptoms experienced by these patients, such as chronic pain, sleep dysfunction, frustration, cognitive impairment, and embarrassment, may contribute to the onset of depression (22). Similar to many chronic conditions, the loss of leisure time spent caring for CRS can be burdensome as well as expensive, leading to heightened anxiety and depression. Moreover, elevated systemic inflammatory cytokine levels seem to contribute to the development of depression. While treatment for comorbid depression may not directly affect the specific quality of life related to CRS, a holistic approach to managing related comorbidities can enhance overall patient care (23).

Most previous epidemiologic studies have employed a case-control design with a vague temporal sequence, making causal inferences difficult. Furthermore, previous observational studies have often struggled with avoiding the interference of confounding risk factors. In contrast, in this study, we leveraged MR methods to elucidate causality while mitigating bias (24, 25). MR analysis is a powerful tool for discerning potential causal relationships between various exposures (risk factors) and disease outcomes (26). Earlier MR analyses have suggested a causal relationship between depression and several chronic diseases, including inflammatory bowel disease, metabolic syndrome, atopic dermatitis, and type 2 diabetes mellitus (25–27).

To date, the causal relationship between CRS and psychiatric disorders remains poorly understood. It is unclear whether depression and anxiety exacerbate CRS symptoms or if these symptoms are a consequence of CRS. Herein we report robust evidence for a bidirectional causal relationship between CRS and depression. As research delves deeper into chronic inflammatory diseases, co-occurring psychiatric disorders are increasingly recognized. A dynamic link is believed to exist between chronic inflammatory diseases and psychiatric disorders, with respiratory inflammation potentially originating at the site of the disease and gradually spreading to other areas (e.g., the central nervous system) (28). Nasal congestion, runny nose, and mucosal lesions are reportedly more severe in CRS patients with comorbid depression than in those without depression (29). This could be because severe nasal symptoms can induce adverse emotional experiences, such as discomfort and lowered self-esteem (30), which can eventually lead to depression. In addition, the nasal congestion or runny nose phenomena involve crucial central mechanisms that merit consideration. These mechanisms are finely tuned by trigeminal autonomic reflexes, whose significance becomes evident, for instance, in specific primary headache disorders (31). Such disorders, interestingly, bear a strong correlation with depression (32). Furthermore, depression can lead to immune dysfunction, causing an increase in inflammatory cytokines in peripheral blood, which may serve as a potential factor contributing to the onset of CRS. However, the specific mechanism behind this remains to be further explored.

Herein our single-cell combined MR analysis led to the identification of two novel genes that may be associated with CRS-associated depression. TCF4 is a basic helix-loop-helix transcription factor involved in early neuronal differentiation, cognitive functions, and immune responses in the brain (33). *TCF4* has been associated with several psychiatric conditions, such as major depressive disorder, schizophrenia, and autism spectrum disorders. Further, its expression at the mRNA and protein levels evidently plays a key role in the pathogenic mechanism of recurrent depression (34). MEF2C is a critical member of the myocyte enhancer factor family, integral to early brain development as well as to neuronal development and electrical activity (35). Mutations or dysfunctions in *MEF2C* have been reported to cause, for example, autism-like symptoms, intellectual deficits, and epilepsy (36). The association between MEF2C and cognitive disorders is extremely similar to the role of MEF2C in autism spectrum disorders and Alzheimer’s disease (37). At present, no study has reported on TCF4 and MEF2C in CRS-related research. We aim to conduct further investigations to comprehensively explore the role of TCF4B and MEF2C in CRS-associated depression.

This study has several strengths. It represents the first bidirectional MR analysis exploring the causal relationship between depression and CRS. Our study design is the closest approximation to a randomized controlled trial, ensuring random allocation based on genotype. By avoiding the limitations of conventional observational studies, such as confounding factors and reverse causality, MR analysis offers a more accurate assessment of causality. Further, we leveraged large sample sizes from the included studies as well as IVs closely related to depression. MR-Egger analysis suggested that all observed causal relationships were unaffected by directional pleiotropy. We also performed sensitivity analyses to determine the impact of pleiotropy on causality estimates, and our results remained robust throughout these various tests.

Some limitations should also be acknowledged. First, all participants included in this study were from Europe, necessitating further research to confirm the generalizability of our findings to other populations. Second, although MR analysis has been demonstrated to be a powerful approach to evaluate causality, its results require validation through additional studies incorporating experimental evidence. Finally, data related to CRS subtypes, disease severity, and comorbidities were not available in the utilized databases, limiting our exploration of factors influencing depression in CRS. CRS manifests in diverse phenotypes that exhibit distinct biological characteristics. Specifically, type 2 CRS (characterized by the presence of polyps) differs significantly from non-type 2 CRS in terms of its immune mechanisms and underlying genetic factors (38). That limits the application of the results presented in this study, especially in regard to cells lines analysis (only CRS with polyps included).

## 5 Conclusions

To summarize, our MR analysis revealed a bidirectional causal relationship between depression and CRS. TCF4 and MEF2C are potential therapeutic targets for CRS with depression. Further studies are warranted to validate our findings. Future evidence from more randomized controlled trials and basic experimental studies can further enhance our understanding of the role of depression in CRS prevention and treatment.

## Data availability statement

The original contributions presented in the study are included in the article/**Supplementary Material**. Further inquiries can be directed to the corresponding authors.

## Author contributions

FZ: Writing – original draft, Methodology, Formal Analysis, Data curation, Conceptualization. YY: Writing – original draft, Software, Investigation, Formal Analysis, Data curation. JL: Writing – original draft, Supervision, Software. YJ: Writing – original draft, Software, Methodology, Investigation. TZ: Writing – review & editing, Visualization, Validation, Supervision, Resources, Project administration, Conceptualization. GY: Writing – review & editing, Visualization, Validation, Supervision, Resources, Project administration, Funding acquisition.
