# Supplementary figures and images for: Mendelian randomization and single-cell expression analyses identify the causal relationship between depression and chronic rhinosinusitis
Source: Front Psychiatry. 2024 May 16;15:1342376. doi: 10.3389/fpsyt.2024.1342376 (PMC11140484; doi:10.3389/fpsyt.2024.1342376)

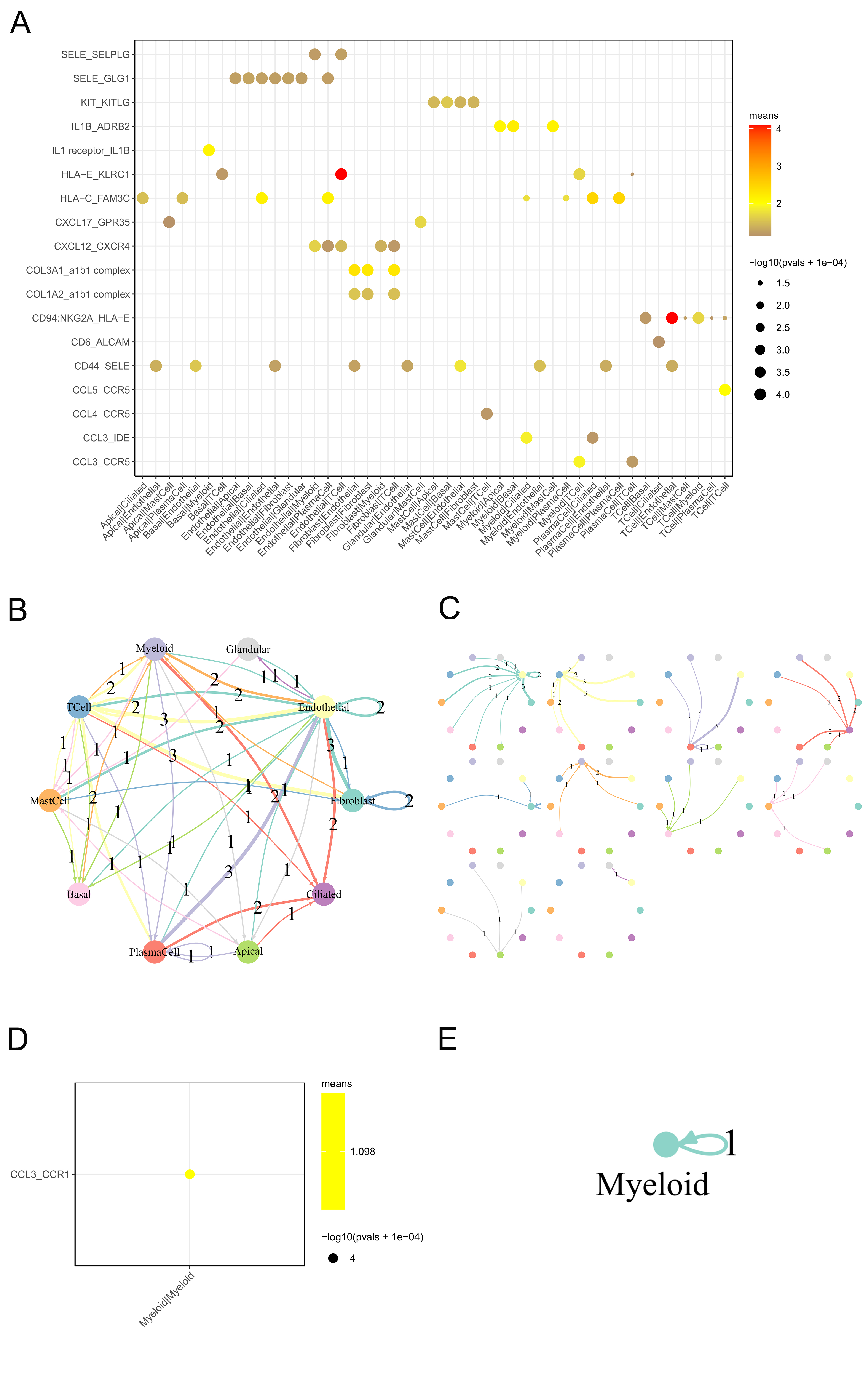

Supplement: Supplementary file 1 [file DataSheet_1.zip › Figure S1.JPEG]
